# Supplementary material for: Neuroimaging Approach: Effects of Hot and Cold Germinated Wheat Beverages on Electroencephalographic (EEG) Activity of the Human Brain
Source: Foods. 2023 Sep 20;12(18):3493. doi: 10.3390/foods12183493 (PMC10527878; doi:10.3390/foods12183493)
Supplement: Supplementary file 1 [file foods-12-03493-s001.zip › foods-2604095-supplementary.pdf]

Supplementary

Table S1. Changes of brain waves (*p* value) after consumption of wheat beverages (HB and CB) determined by *t*-test for each channel.

Ch.	***p*-values (*t*-test)**							
	Delta (1 – 4 Hz)		Theta (4 – 8 Hz)		Alpha (8 – 12 Hz)		Beta (12 – 25 Hz)	
	HB_1	CB_1	HB_1	CB_1	HB_1	CB_1	HB_1	CB_1
FP1	0.423	0.926	0.051	0.959	0.277	0.222	0.004	0.237
FP2	0.870	0.811	0.023	0.961	0.346	0.115	0.071	0.422
F3	0.193	0.947	0.064	0.602	0.934	0.154	0.175	0.141
F4	0.275	0.388	0.070	0.630	0.774	0.183	0.201	0.222
F7	0.041	0.865	0.040	0.984	0.855	0.111	0.059	0.117
F8	0.339	0.339	0.094	0.550	0.650	0.183	0.045	0.568
Fz	0.408	0.399	0.037	0.775	0.310	0.112	0.060	0.059
C3	0.171	0.778	0.089	0.387	0.869	0.141	0.884	0.048
C4	0.121	0.256	0.115	0.257	0.735	0.136	0.740	0.264
Cz	0.294	0.476	0.056	0.944	0.306	0.337	0.378	0.242
T3	0.310	0.775	0.722	0.661	0.468	0.104	0.247	0.271
T4	0.070	0.194	0.475	0.091	0.659	0.462	0.070	0.372
T5	0.373	0.621	0.282	0.932	0.333	0.694	0.478	0.068
T6	0.338	0.553	0.101	0.278	0.709	0.176	0.679	0.938
P3	0.336	0.638	0.137	0.313	0.884	0.146	0.773	0.208
P4	0.226	0.706	0.069	0.439	0.604	0.136	0.617	0.466
Pz	0.322	0.446	0.107	0.927	0.632	0.212	0.549	0.566
O1	0.266	0.634	0.043	0.775	0.731	0.220	0.415	0.595
O2	0.401	0.286	0.216	0.285	0.473	0.170	0.549	0.422

HB, hot germinate-wheat beverage 0.8 g/100 mL; CB, cold germinated-wheat beverage 25 g/L.

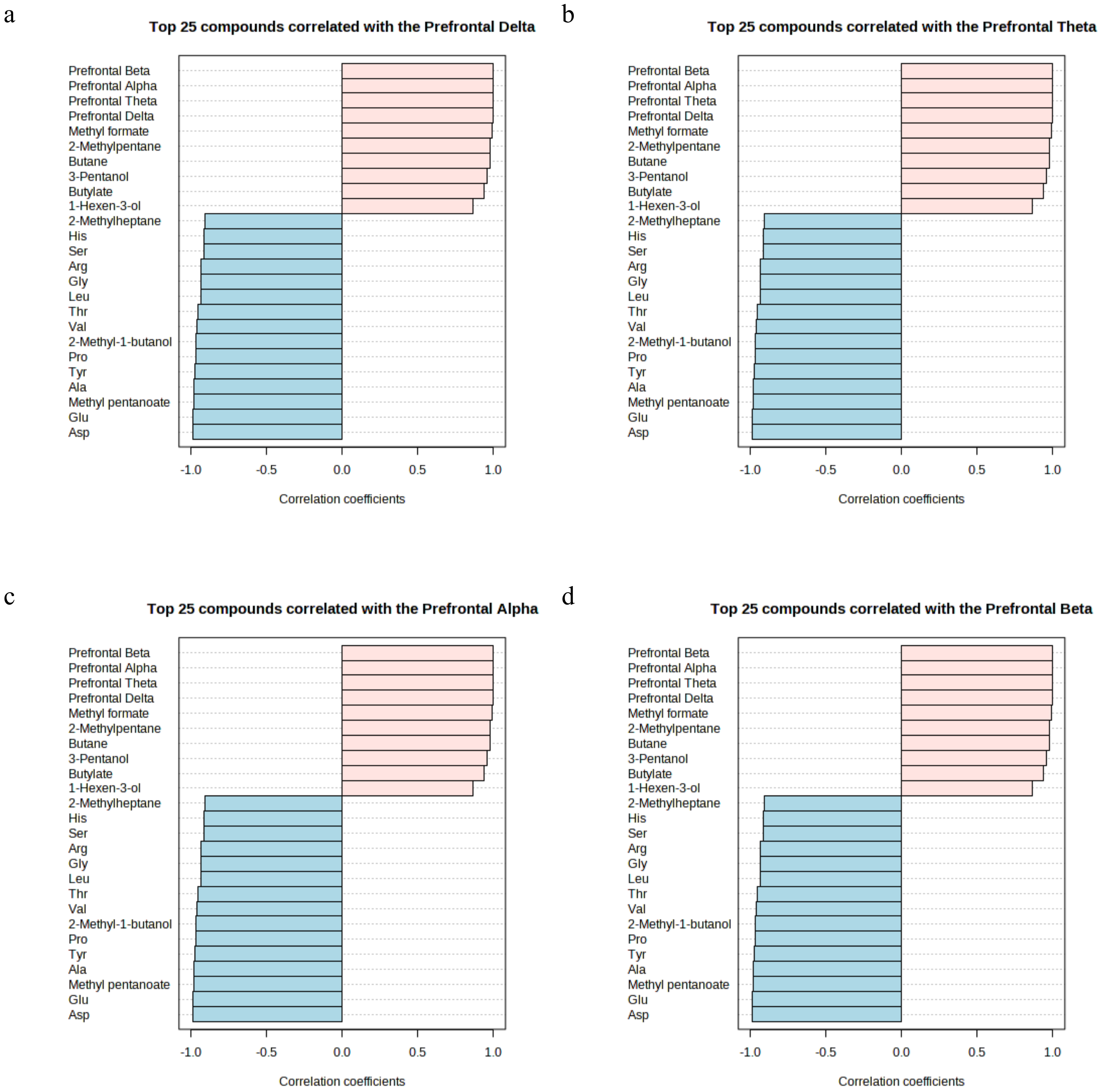
a
Top 25 compounds correlated with the Prefrontal Delta
Prefrontal Beta
Prefrontal Alpha
Prefrontal Theta
Prefrontal Delta
Methyl formate
2-Methylpentane
Butane
3-Pentanol
Butylate
1-Hexen-3-ol
2-Methylheptane
His
Ser
Arg
Gly
Leu
Thr
Val
2-Methyl-1-butanol
Pro
Tyr
Ala
Methyl pentanoate
Glu
Asp
-1.0
-0.5
0.0
0.5
1.0
Correlation coefficients
b
Top 25 compounds correlated with the Prefrontal Theta
Prefrontal Beta
Prefrontal Alpha
Prefrontal Theta
Prefrontal Delta
Methyl formate
2-Methylpentane
Butane
3-Pentanol
Butylate
1-Hexen-3-ol
2-Methylheptane
His
Ser
Arg
Gly
Leu
Thr
Val
2-Methyl-1-butanol
Pro
Tyr
Ala
Methyl pentanoate
Glu
Asp
-1.0
-0.5
0.0
0.5
1.0
Correlation coefficients
c
Top 25 compounds correlated with the Prefrontal Alpha
Prefrontal Beta
Prefrontal Alpha
Prefrontal Theta
Prefrontal Delta
Methyl formate
2-Methylpentane
Butane
3-Pentanol
Butylate
1-Hexen-3-ol
2-Methylheptane
His
Ser
Arg
Gly
Leu
Thr
Val
2-Methyl-1-butanol
Pro
Tyr
Ala
Methyl pentanoate
Glu
Asp
-1.0
-0.5
0.0
0.5
1.0
Correlation coefficients
d
Top 25 compounds correlated with the Prefrontal Beta
Prefrontal Beta
Prefrontal Alpha
Prefrontal Theta
Prefrontal Delta
Methyl formate
2-Methylpentane
Butane
3-Pentanol
Butylate
1-Hexen-3-ol
2-Methylheptane
His
Ser
Arg
Gly
Leu
Thr
Val
2-Methyl-1-butanol
Pro
Tyr
Ala
Methyl pentanoate
Glu
Asp
-1.0
-0.5
0.0
0.5
1.0
Correlation coefficients

e

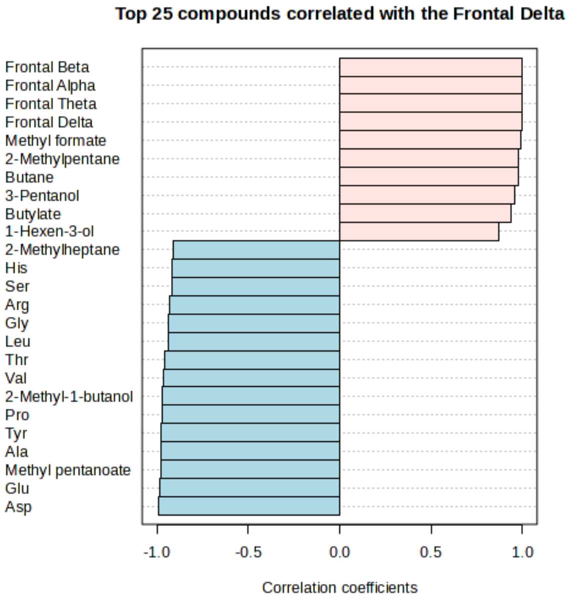
Top 25 compounds correlated with the Frontal Delta
Frontal Beta
Frontal Alpha
Frontal Theta
Frontal Delta
Methyl formate
2-Methylpentane
Butane
3-Pentanol
Butylate
1-Hexen-3-ol
2-Methylheptane
His
Ser
Arg
Gly
Leu
Thr
Val
2-Methyl-1-butanol
Pro
Tyr
Ala
Methyl pentanoate
Glu
Asp
-1.0
-0.5
0.0
0.5
1.0
Correlation coefficients

f

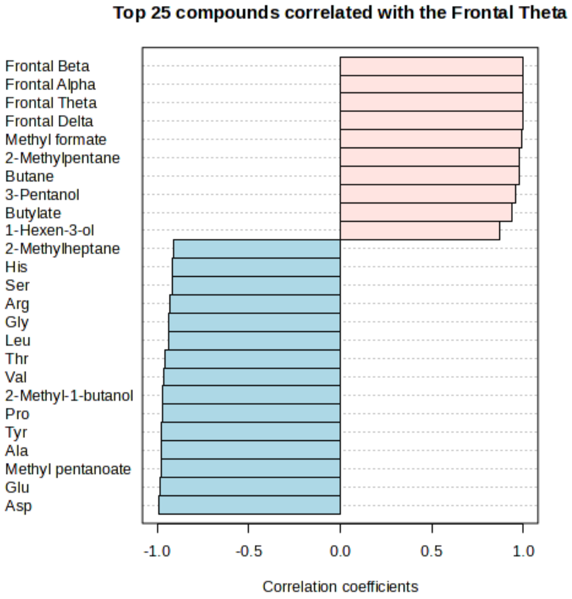
Top 25 compounds correlated with the Frontal Theta
Frontal Beta
Frontal Alpha
Frontal Theta
Frontal Delta
Methyl formate
2-Methylpentane
Butane
3-Pentanol
Butylate
1-Hexen-3-ol
2-Methylheptane
His
Ser
Arg
Gly
Leu
Thr
Val
2-Methyl-1-butanol
Pro
Tyr
Ala
Methyl pentanoate
Glu
Asp
-1.0
-0.5
0.0
0.5
1.0
Correlation coefficients

g

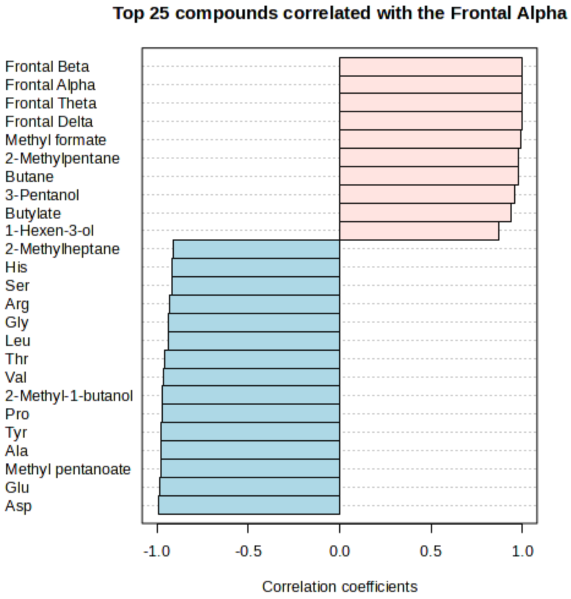
Top 25 compounds correlated with the Frontal Alpha
Frontal Beta
Frontal Alpha
Frontal Theta
Frontal Delta
Methyl formate
2-Methylpentane
Butane
3-Pentanol
Butylate
1-Hexen-3-ol
2-Methylheptane
His
Ser
Arg
Gly
Leu
Thr
Val
2-Methyl-1-butanol
Pro
Tyr
Ala
Methyl pentanoate
Glu
Asp
-1.0
-0.5
0.0
0.5
1.0
Correlation coefficients

h

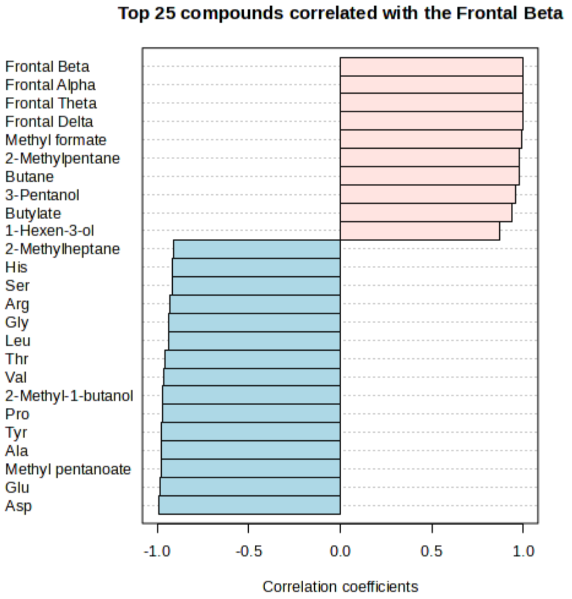
Top 25 compounds correlated with the Frontal Beta
Frontal Beta
Frontal Alpha
Frontal Theta
Frontal Delta
Methyl formate
2-Methylpentane
Butane
3-Pentanol
Butylate
1-Hexen-3-ol
2-Methylheptane
His
Ser
Arg
Gly
Leu
Thr
Val
2-Methyl-1-butanol
Pro
Tyr
Ala
Methyl pentanoate
Glu
Asp
-1.0
-0.5
0.0
0.5
1.0
Correlation coefficients

i

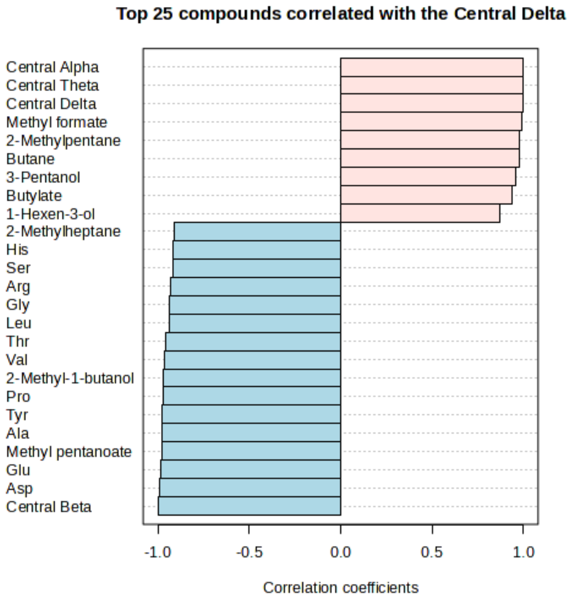
Top 25 compounds correlated with the Central Delta
Central Alpha
Central Theta
Central Delta
Methyl formate
2-Methylpentane
Butane
3-Pentanol
Butylate
1-Hexen-3-ol
2-Methylheptane
His
Ser
Arg
Gly
Leu
Thr
Val
2-Methyl-1-butanol
Pro
Tyr
Ala
Methyl pentanoate
Glu
Asp
Central Beta
-1.0
-0.5
0.0
0.5
1.0
Correlation coefficients

j

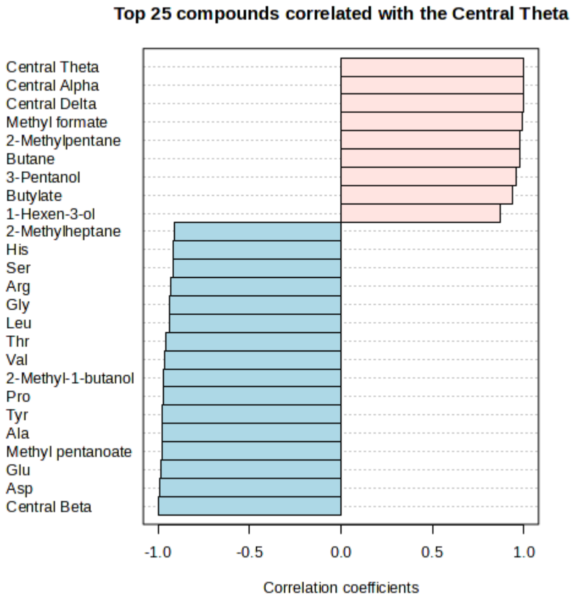
Top 25 compounds correlated with the Central Theta
Central Theta
Central Alpha
Central Delta
Methyl formate
2-Methylpentane
Butane
3-Pentanol
Butylate
1-Hexen-3-ol
2-Methylheptane
His
Ser
Arg
Gly
Leu
Thr
Val
2-Methyl-1-butanol
Pro
Tyr
Ala
Methyl pentanoate
Glu
Asp
Central Beta
-1.0
-0.5
0.0
0.5
1.0
Correlation coefficients

k

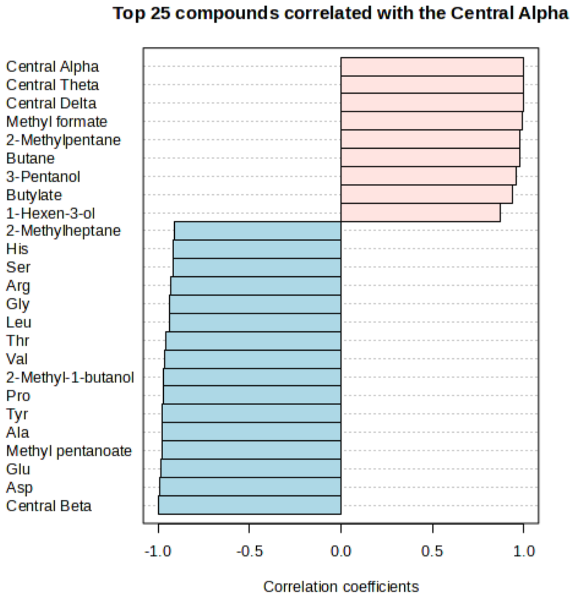
Top 25 compounds correlated with the Central Alpha
Central Alpha
Central Theta
Central Delta
Methyl formate
2-Methylpentane
Butane
3-Pentanol
Butylate
1-Hexen-3-ol
2-Methylheptane
His
Ser
Arg
Gly
Leu
Thr
Val
2-Methyl-1-butanol
Pro
Tyr
Ala
Methyl pentanoate
Glu
Asp
Central Beta
-1.0
-0.5
0.0
0.5
1.0
Correlation coefficients

l

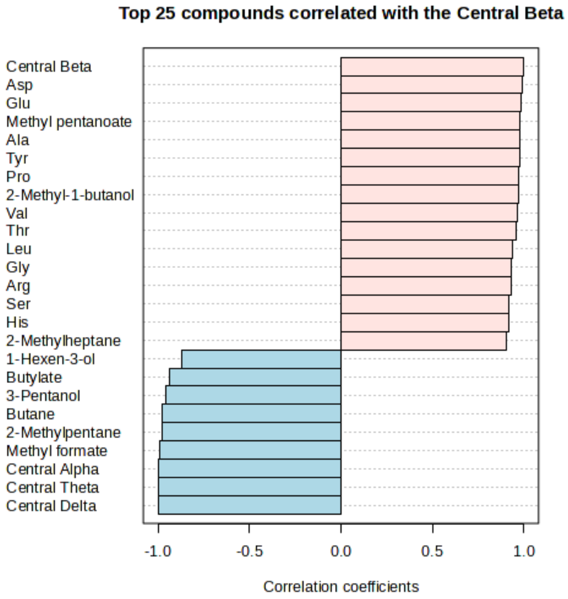
Top 25 compounds correlated with the Central Beta
Central Beta
Asp
Glu
Methyl pentanoate
Ala
Tyr
Pro
2-Methyl-1-butanol
Val
Thr
Leu
Gly
Arg
Ser
His
2-Methylheptane
1-Hexen-3-ol
Butylate
3-Pentanol
Butane
2-Methylpentane
Methyl formate
Central Alpha
Central Theta
Central Delta
-1.0
-0.5
0.0
0.5
1.0
Correlation coefficients

m

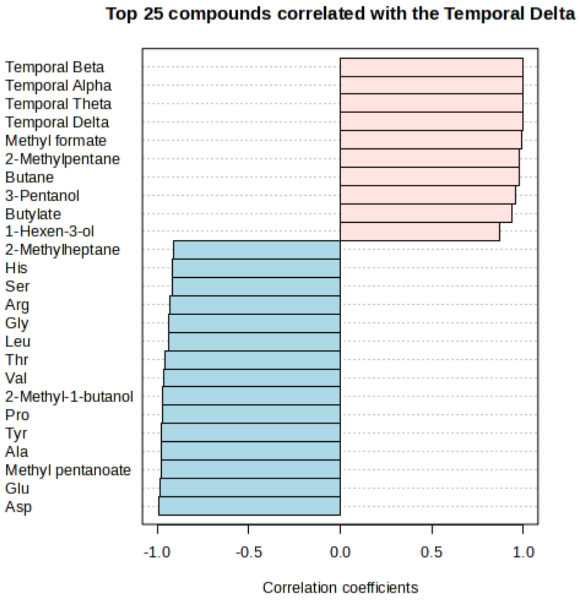

Top 25 compounds correlated with the Temporal Delta
Temporal Beta
Temporal Alpha
Temporal Theta
Temporal Delta
Methyl formate
2-Methylpentane
Butane
3-Pentanol
Butylate
1-Hexen-3-ol
2-Methylheptane
His
Ser
Arg
Gly
Leu
Thr
Val
2-Methyl-1-butanol
Pro
Tyr
Ala
Methyl pentanoate
Glu
Asp
-1.0
-0.5
0.0
0.5
1.0
Correlation coefficients

n

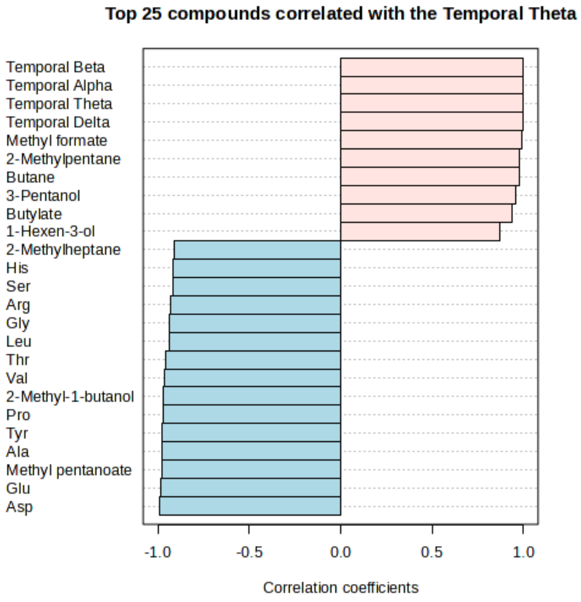

Top 25 compounds correlated with the Temporal Theta
Temporal Beta
Temporal Alpha
Temporal Theta
Temporal Delta
Methyl formate
2-Methylpentane
Butane
3-Pentanol
Butylate
1-Hexen-3-ol
2-Methylheptane
His
Ser
Arg
Gly
Leu
Thr
Val
2-Methyl-1-butanol
Pro
Tyr
Ala
Methyl pentanoate
Glu
Asp
-1.0
-0.5
0.0
0.5
1.0
Correlation coefficients

o

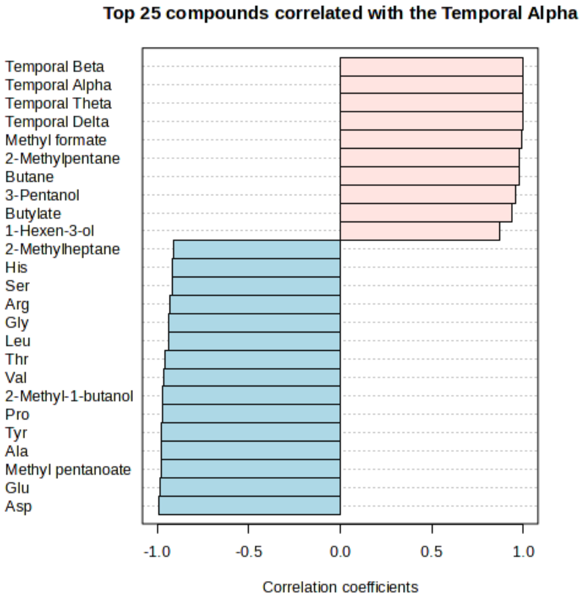

Top 25 compounds correlated with the Temporal Alpha
Temporal Beta
Temporal Alpha
Temporal Theta
Temporal Delta
Methyl formate
2-Methylpentane
Butane
3-Pentanol
Butylate
1-Hexen-3-ol
2-Methylheptane
His
Ser
Arg
Gly
Leu
Thr
Val
2-Methyl-1-butanol
Pro
Tyr
Ala
Methyl pentanoate
Glu
Asp
-1.0
-0.5
0.0
0.5
1.0
Correlation coefficients

p

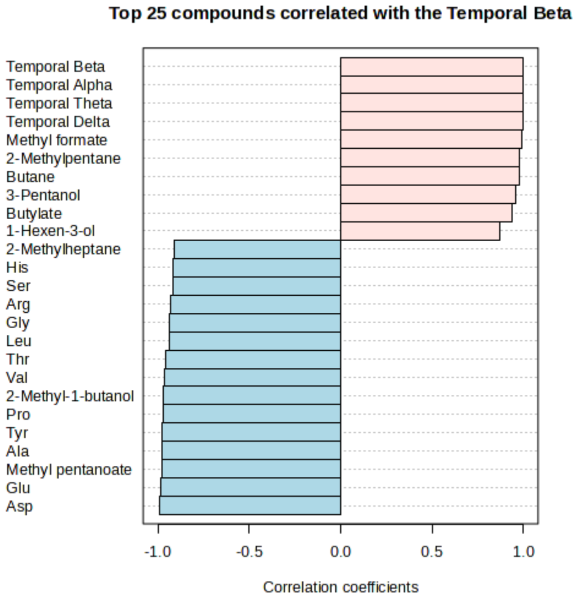

Top 25 compounds correlated with the Temporal Beta
Temporal Beta
Temporal Alpha
Temporal Theta
Temporal Delta
Methyl formate
2-Methylpentane
Butane
3-Pentanol
Butylate
1-Hexen-3-ol
2-Methylheptane
His
Ser
Arg
Gly
Leu
Thr
Val
2-Methyl-1-butanol
Pro
Tyr
Ala
Methyl pentanoate
Glu
Asp
-1.0
-0.5
0.0
0.5
1.0
Correlation coefficients

q

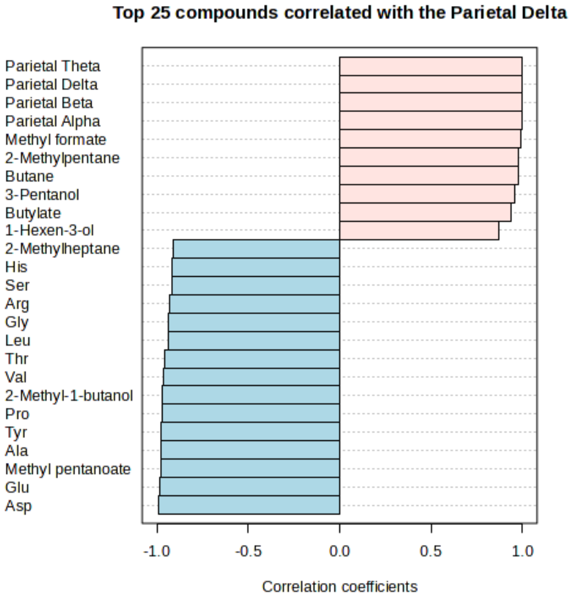
Top 25 compounds correlated with the Parietal Delta
Parietal Theta
Parietal Delta
Parietal Beta
Parietal Alpha
Methyl formate
2-Methylpentane
Butane
3-Pentanol
Butylate
1-Hexen-3-ol
2-Methylheptane
His
Ser
Arg
Gly
Leu
Thr
Val
2-Methyl-1-butanol
Pro
Tyr
Ala
Methyl pentanoate
Glu
Asp
-1.0
-0.5
0.0
0.5
1.0
Correlation coefficients

r

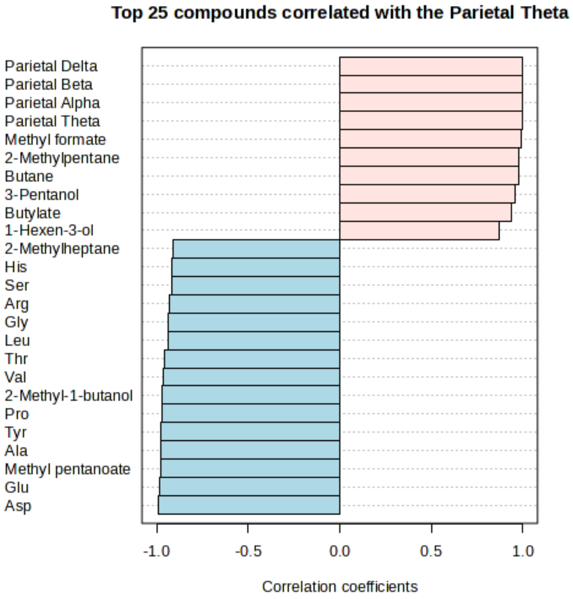
Top 25 compounds correlated with the Parietal Theta
Parietal Delta
Parietal Beta
Parietal Alpha
Parietal Theta
Methyl formate
2-Methylpentane
Butane
3-Pentanol
Butylate
1-Hexen-3-ol
2-Methylheptane
His
Ser
Arg
Gly
Leu
Thr
Val
2-Methyl-1-butanol
Pro
Tyr
Ala
Methyl pentanoate
Glu
Asp
-1.0
-0.5
0.0
0.5
1.0
Correlation coefficients

s

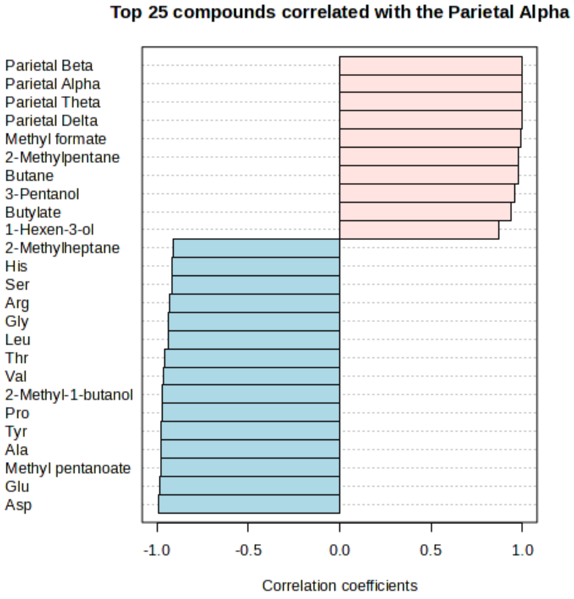
Top 25 compounds correlated with the Parietal Alpha
Parietal Beta
Parietal Alpha
Parietal Theta
Parietal Delta
Methyl formate
2-Methylpentane
Butane
3-Pentanol
Butylate
1-Hexen-3-ol
2-Methylheptane
His
Ser
Arg
Gly
Leu
Thr
Val
2-Methyl-1-butanol
Pro
Tyr
Ala
Methyl pentanoate
Glu
Asp
-1.0
-0.5
0.0
0.5
1.0
Correlation coefficients

t

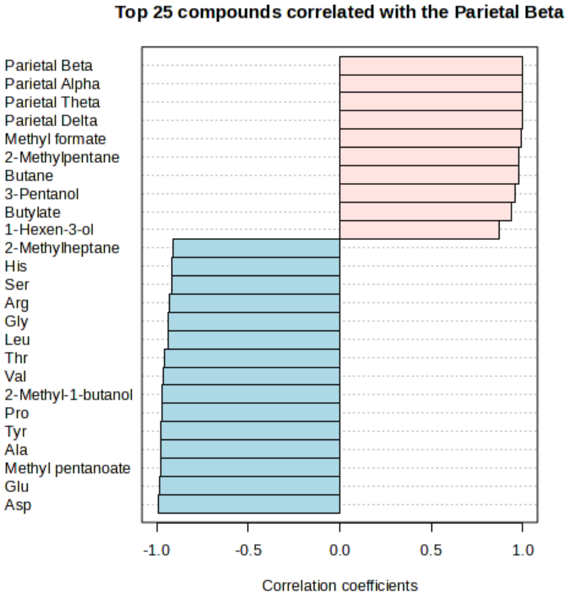
Top 25 compounds correlated with the Parietal Beta
Parietal Beta
Parietal Alpha
Parietal Theta
Parietal Delta
Methyl formate
2-Methylpentane
Butane
3-Pentanol
Butylate
1-Hexen-3-ol
2-Methylheptane
His
Ser
Arg
Gly
Leu
Thr
Val
2-Methyl-1-butanol
Pro
Tyr
Ala
Methyl pentanoate
Glu
Asp
-1.0
-0.5
0.0
0.5
1.0
Correlation coefficients

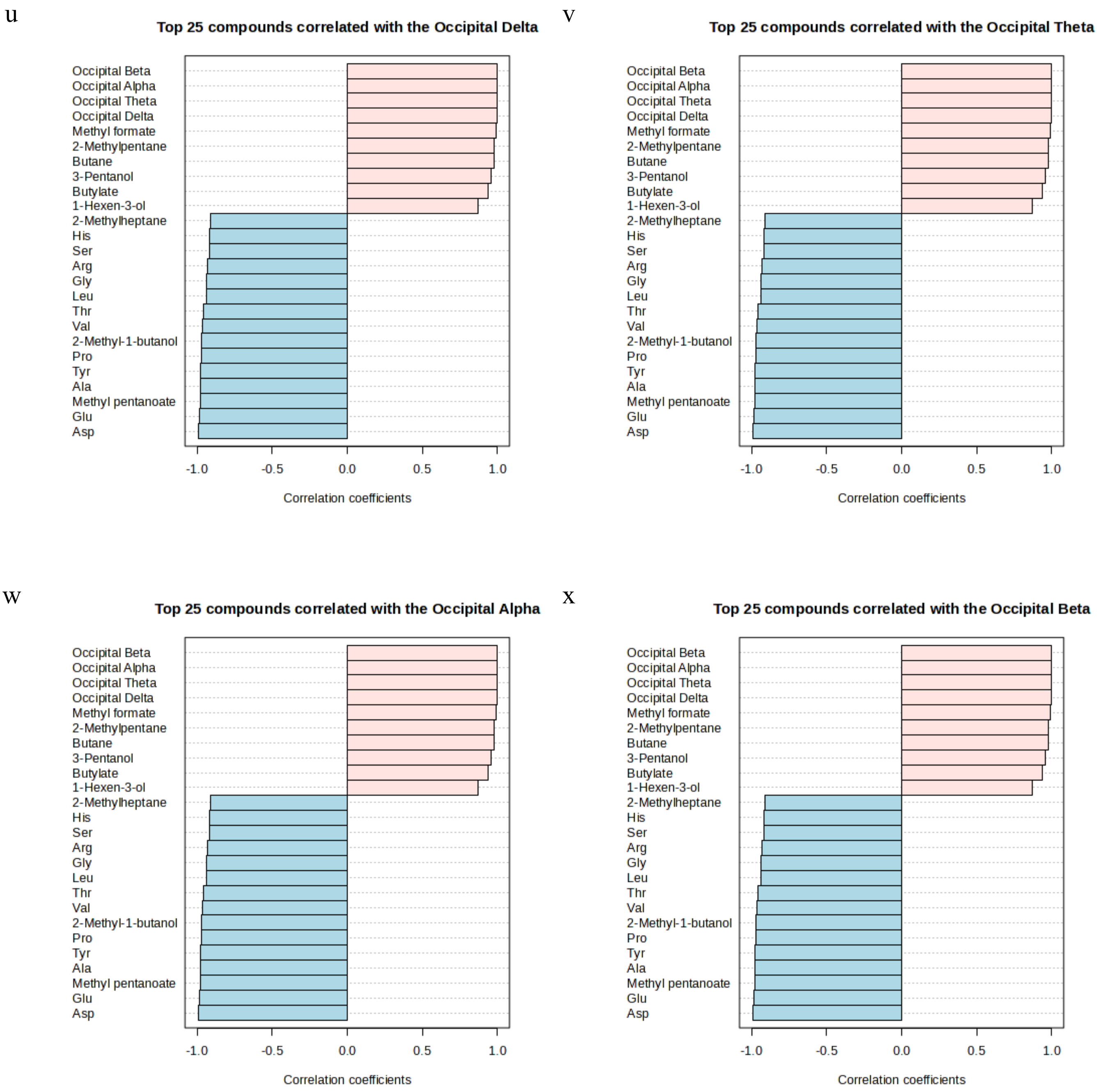

Figure S1. Correlation pattern of amino acids and volatiles compounds contained in hot and cold germinated wheat beverages with changes of EEG waves over cortical subregions.

Top 25 compounds correlated with changes of delta, theta, alpha, and beta bands at the prefrontal cortex (a, b, c, d), frontal cortex (e, f, g, h), central (i, j, k, l), temporal (m, n, o, p), parietal (q, r, s, t), and occipital (u, v, w, x), respectively. Ala, alanine; Arg, arginine; Asp, aspartic acid; Glu, glutamic acid; Gly, glycine; His, histidine; Ile, isoleucine; Leu, leucine; Lys, lysine; Met, methionine; Phe, phenylalanine; Pro, proline; Ser, serine; Thr, threonine; Tys, tyrosine; Val, valine.

Data were analyzed by Pearson r distance measure through Metaboanalyst.

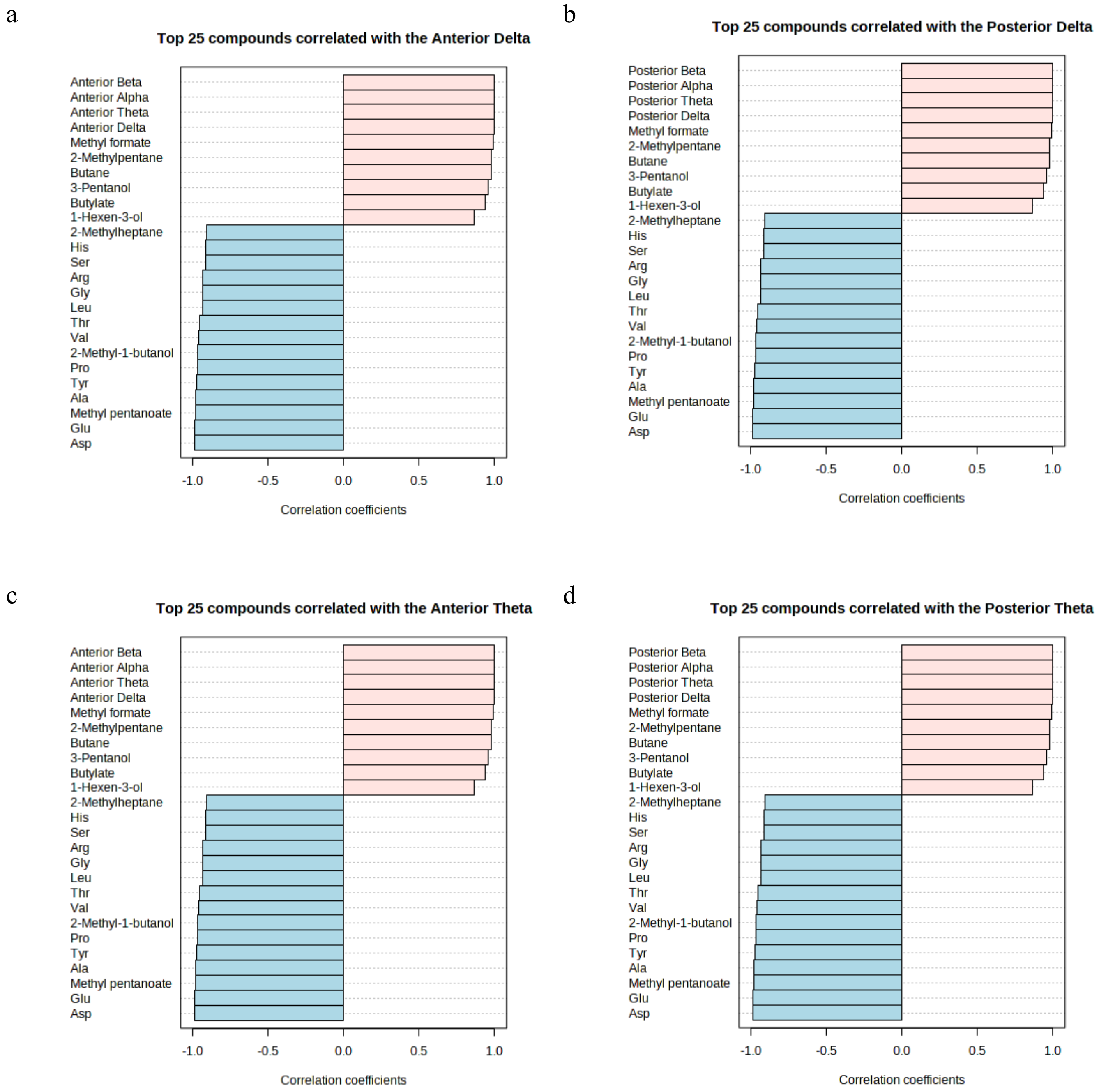
a
Top 25 compounds correlated with the Anterior Delta
Anterior Beta
Anterior Alpha
Anterior Theta
Anterior Delta
Methyl formate
2-Methylpentane
Butane
3-Pentanol
Butylate
1-Hexen-3-ol
2-Methylheptane
His
Ser
Arg
Gly
Leu
Thr
Val
2-Methyl-1-butanol
Pro
Tyr
Ala
Methyl pentanoate
Glu
Asp
-1.0 -0.5 0.0 0.5 1.0
Correlation coefficients
b
Top 25 compounds correlated with the Posterior Delta
Posterior Beta
Posterior Alpha
Posterior Theta
Posterior Delta
Methyl formate
2-Methylpentane
Butane
3-Pentanol
Butylate
1-Hexen-3-ol
2-Methylheptane
His
Ser
Arg
Gly
Leu
Thr
Val
2-Methyl-1-butanol
Pro
Tyr
Ala
Methyl pentanoate
Glu
Asp
-1.0 -0.5 0.0 0.5 1.0
Correlation coefficients
c
Top 25 compounds correlated with the Anterior Theta
Anterior Beta
Anterior Alpha
Anterior Theta
Anterior Delta
Methyl formate
2-Methylpentane
Butane
3-Pentanol
Butylate
1-Hexen-3-ol
2-Methylheptane
His
Ser
Arg
Gly
Leu
Thr
Val
2-Methyl-1-butanol
Pro
Tyr
Ala
Methyl pentanoate
Glu
Asp
-1.0 -0.5 0.0 0.5 1.0
Correlation coefficients
d
Top 25 compounds correlated with the Posterior Theta
Posterior Beta
Posterior Alpha
Posterior Theta
Posterior Delta
Methyl formate
2-Methylpentane
Butane
3-Pentanol
Butylate
1-Hexen-3-ol
2-Methylheptane
His
Ser
Arg
Gly
Leu
Thr
Val
2-Methyl-1-butanol
Pro
Tyr
Ala
Methyl pentanoate
Glu
Asp
-1.0 -0.5 0.0 0.5 1.0
Correlation coefficients

e

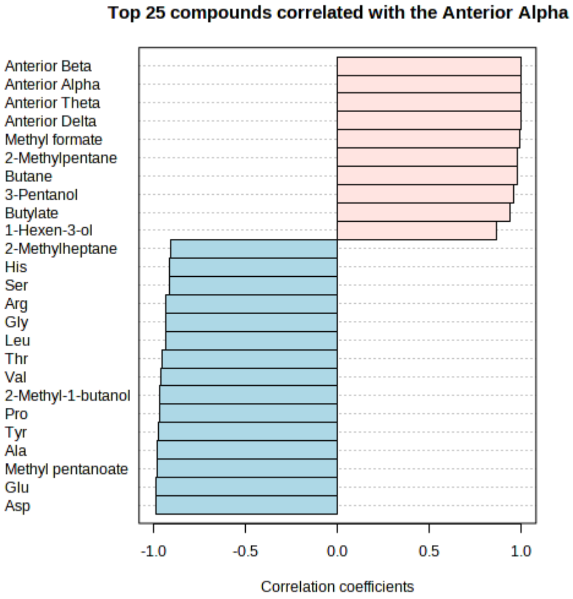

f

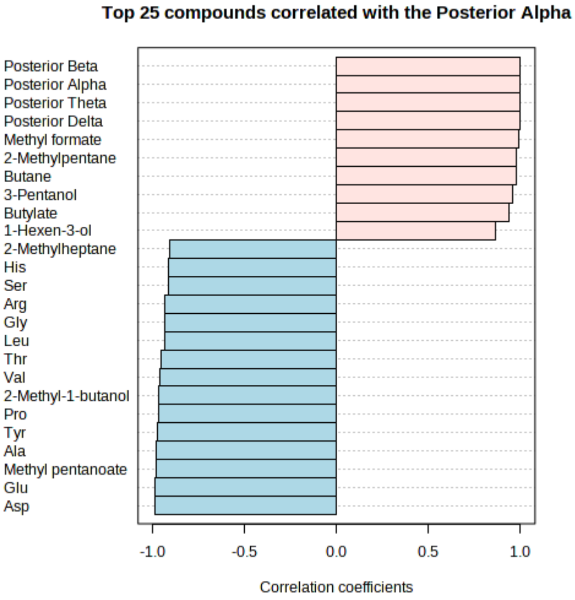

g

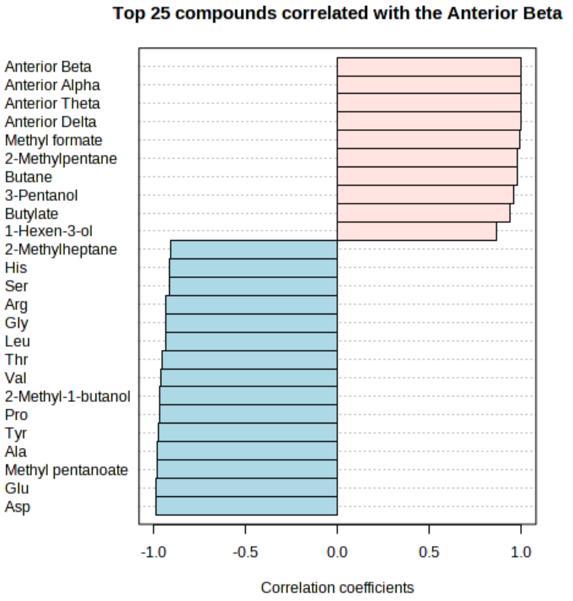

h

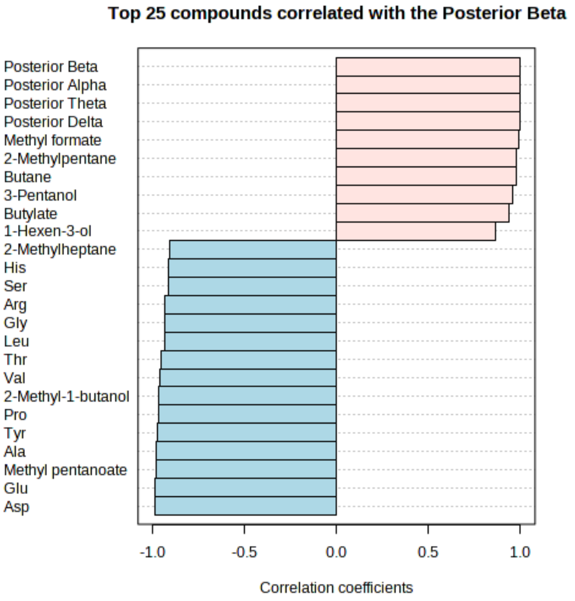

Figure S2. Correlation pattern of amino acids and volatiles compounds contained in hot and cold germinated wheat beverages with changes of EEG waves over anterior and posterior subregions.

Top 25 compounds correlated with changes of delta over the anterior subregions (a), and posterior subregions (b), theta over the anterior subregions (c), and posterior subregions (d), alpha over the anterior subregions (e), and posterior subregions (f), and beta over the anterior subregions (g), and posterior subregions (h), respectively. Ala, alanine; Arg, arginine; Asp, aspartic acid; Glu, glutamic acid; Gly, glycine; His, histidine; Ile, isoleucine; Leu, leucine; Lys, lysine; Met, methionine; Phe, phenylalanine; Pro, proline; Ser, serine; Thr, threonine; Tys, tyrosine; Val, valine.

Data were analyzed by Pearson r distance measure through Metaboanalyst.

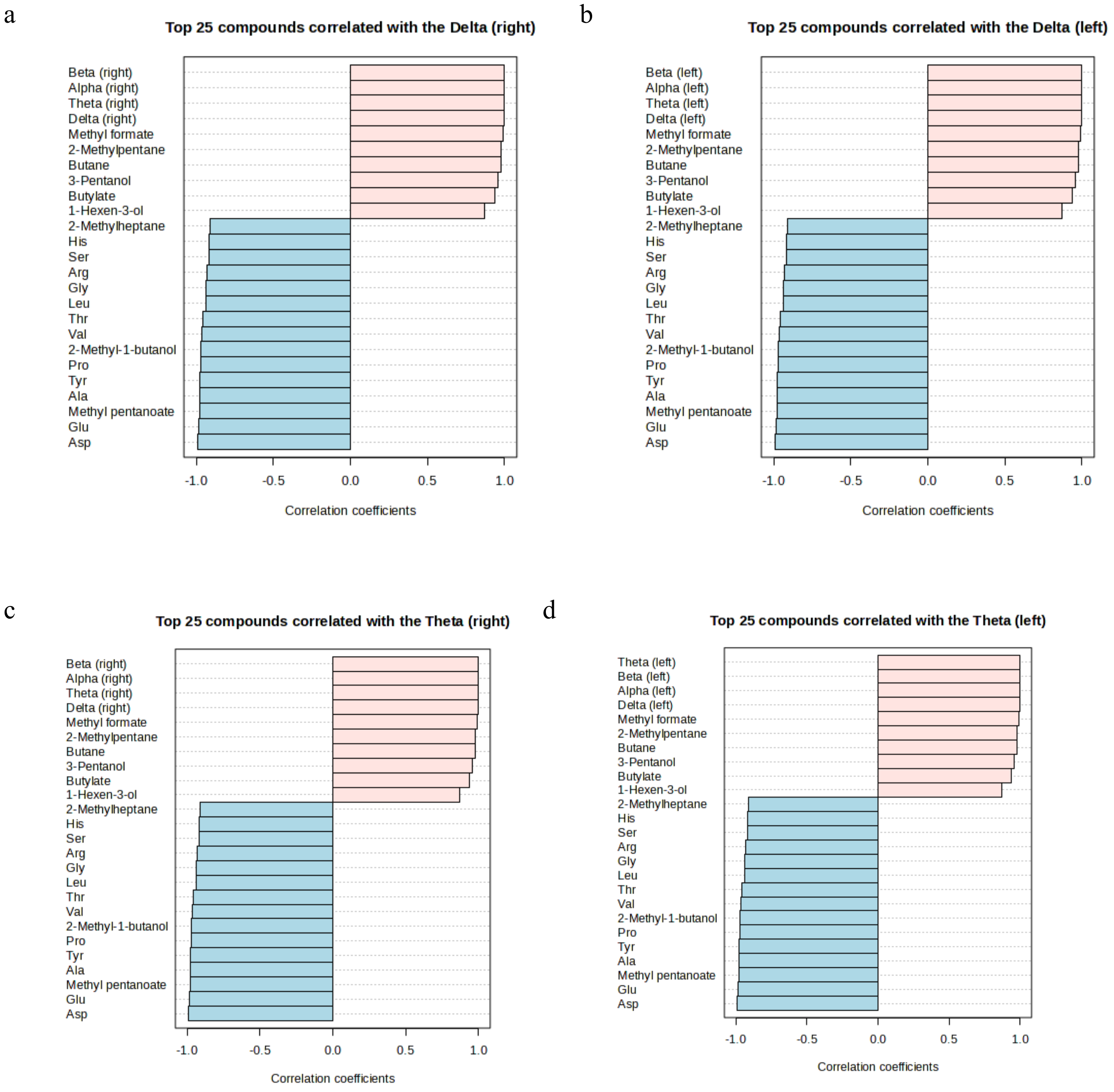
a
Top 25 compounds correlated with the Delta (right)
Beta (right)
Alpha (right)
Theta (right)
Delta (right)
Methyl formate
2-Methylpentane
Butane
3-Pentanol
Butylate
1-Hexen-3-ol
2-Methylheptane
His
Ser
Arg
Gly
Leu
Thr
Val
2-Methyl-1-butanol
Pro
Tyr
Ala
Methyl pentanoate
Glu
Asp
-1.0 -0.5 0.0 0.5 1.0
Correlation coefficients
b
Top 25 compounds correlated with the Delta (left)
Beta (left)
Alpha (left)
Theta (left)
Delta (left)
Methyl formate
2-Methylpentane
Butane
3-Pentanol
Butylate
1-Hexen-3-ol
2-Methylheptane
His
Ser
Arg
Gly
Leu
Thr
Val
2-Methyl-1-butanol
Pro
Tyr
Ala
Methyl pentanoate
Glu
Asp
-1.0 -0.5 0.0 0.5 1.0
Correlation coefficients
c
Top 25 compounds correlated with the Theta (right)
Beta (right)
Alpha (right)
Theta (right)
Delta (right)
Methyl formate
2-Methylpentane
Butane
3-Pentanol
Butylate
1-Hexen-3-ol
2-Methylheptane
His
Ser
Arg
Gly
Leu
Thr
Val
2-Methyl-1-butanol
Pro
Tyr
Ala
Methyl pentanoate
Glu
Asp
-1.0 -0.5 0.0 0.5 1.0
Correlation coefficients
d
Top 25 compounds correlated with the Theta (left)
Theta (left)
Beta (left)
Alpha (left)
Delta (left)
Methyl formate
2-Methylpentane
Butane
3-Pentanol
Butylate
1-Hexen-3-ol
2-Methylheptane
His
Ser
Arg
Gly
Leu
Thr
Val
2-Methyl-1-butanol
Pro
Tyr
Ala
Methyl pentanoate
Glu
Asp
-1.0 -0.5 0.0 0.5 1.0
Correlation coefficients

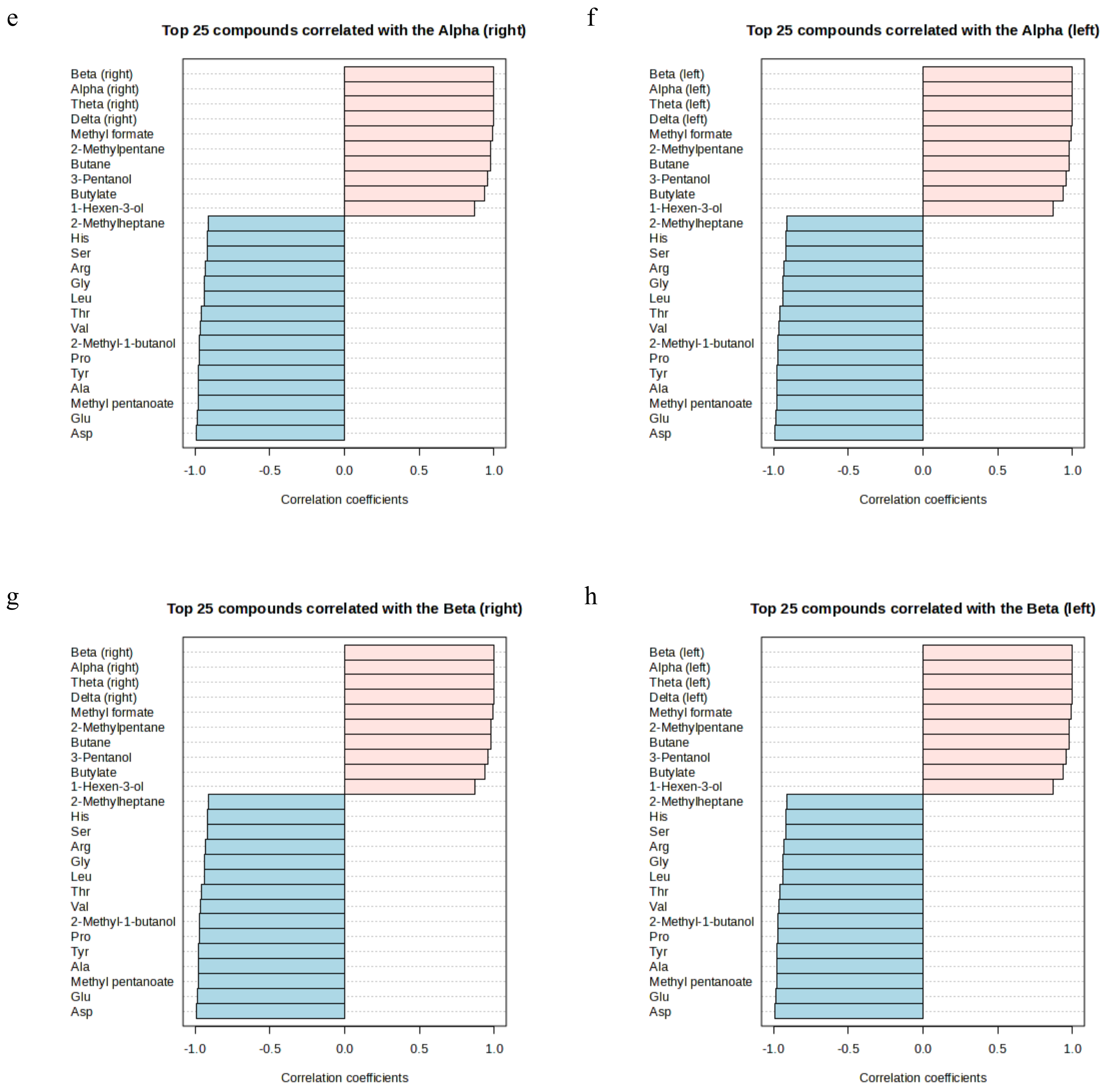

Figure S3. Correlation pattern of amino acids and volatiles compounds contained in hot and cold germinated wheat beverages with changes of EEG waves over left and right hemisphere.

Top 25 compounds correlated with changes of delta over the left-hemisphere (a), and right-hemisphere (b), theta over the left-hemisphere (c), and right-hemisphere (d), alpha over the left-hemisphere (e), and right-hemisphere (f), and beta over the left-hemisphere (g), and right-hemisphere (h), respectively. Ala, alanine; Arg, arginine; Asp, aspartic acid; Glu, glutamic acid; Gly, glycine; His, histidine; Ile, isoleucine; Leu, leucine; Lys, lysine; Met, methionine; Phe, phenylalanine; Pro, proline; Ser, serine; Thr, threonine; Tys, tyrosine; Val, valine.

Data were analyzed by Pearson r distance measure through Metaboanalyst.

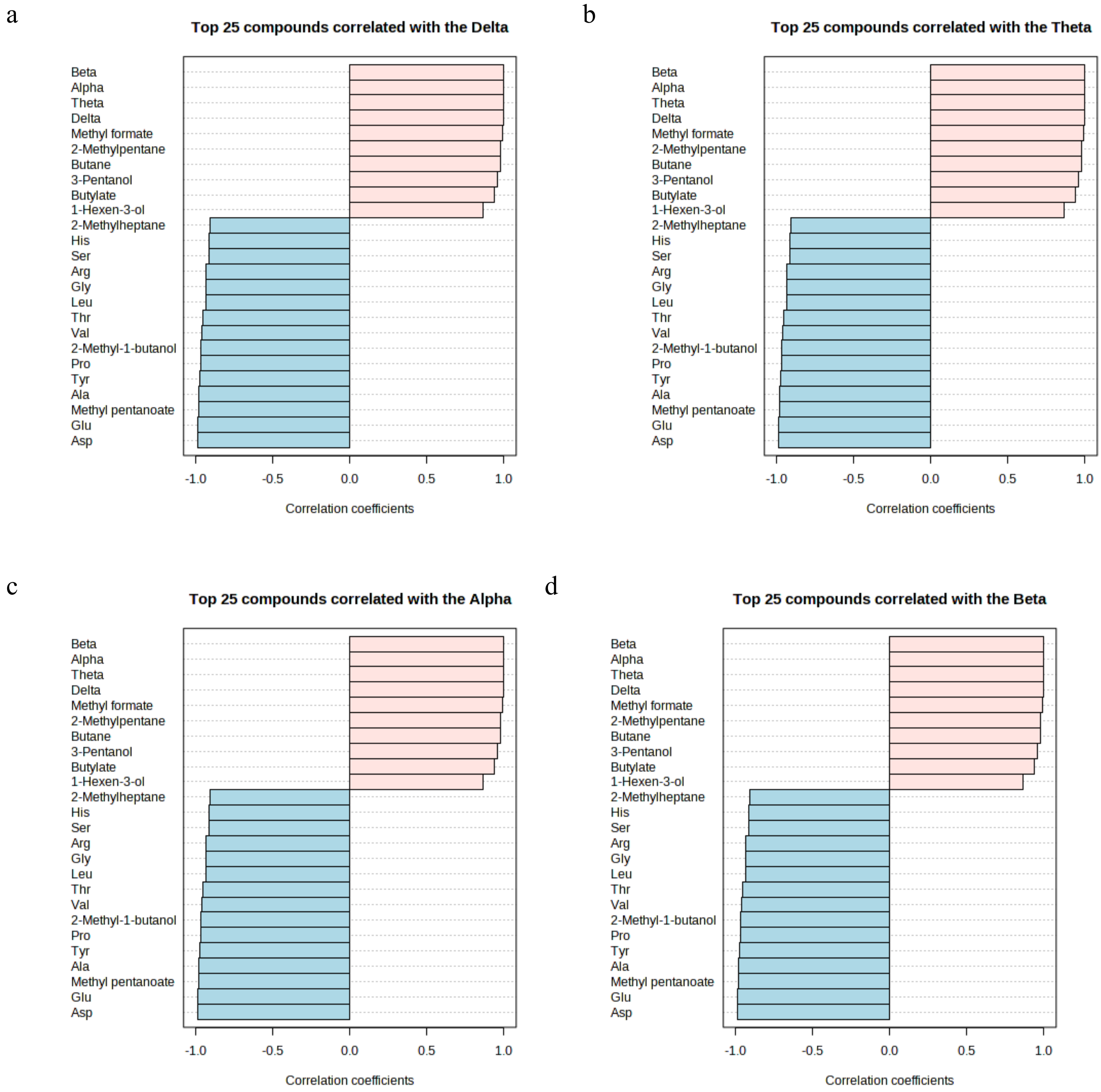

Figure S4. Correlation pattern of amino acids and volatiles compounds contained in hot and cold germinated wheat beverages with changes of EEG waves across whole cortex.

Top 25 compounds correlated with changes of delta (a), theta (b), alpha (c), and beta (d) across the whole cortex. Ala, alanine; Arg, arginine; Asp, aspartic acid; Glu, glutamic acid; Gly, glycine; His, histidine; Ile, isoleucine; Leu, leucine; Lys, lysine; Met, methionine; Phe, phenylalanine; Pro, proline; Ser, serine; Thr, threonine; Tys, tyrosine; Val, valine.

Data were analyzed by Pearson r distance measure through Metaboanalyst.
